# Neck Pain and Symptomatic Hypothyroidism: An Atypical Presentation of Subacute Thyroiditis

**DOI:** 10.7759/cureus.58148

**Published:** 2024-04-12

**Authors:** Mary C Mitchell, Daniel Shults, English Gonzalez

**Affiliations:** 1 Medical School, Edward Via College of Osteopathic Medicine, Auburn, USA; 2 Family Medicine, Christ Health Center, Birmingham, USA

**Keywords:** painful thyroiditis, subacute granulomatous thyroiditis, inflammatory neck pain, clinical hypothyroidism, subacute thyroiditis

## Abstract

Subacute thyroiditis (SAT) is a rare form of thyroid disease characterized by fever, neck pain, and dysregulated thyroid hormone levels. It is caused by the post-viral inflammation and destruction of thyroid follicles. Patients typically present with symptoms of hyperthyroidism, as stored thyroid hormone is released into the blood. In this case, we describe a 34-year-old female who presented to the clinic complaining of neck pain and a headache for two days. She endorsed fatigue, myalgias, dizziness, and constipation but denied any fever. She reported only minimal pain relief with ibuprofen and denied a history of recent illness. On exam, she was afebrile and normotensive. Her physical exam was notable for neck tenderness over the right lobe and isthmus of the thyroid, thyromegaly, and a palpable thyroid nodule. Her complete blood count showed no sign of infection or hematologic abnormality, but her thyroid studies showed an elevated thyroid stimulating hormone of 2.1 mIU/L and a decreased thyroxine (T4) level below 0.01 ng/dL. The laboratory results, history, and physical exam led to the diagnosis of the hypothyroid stage of subacute thyroiditis. She was initially treated with ibuprofen 600mg without resolution of her symptoms. She was then treated with prednisone 40mg with symptom relief. This case highlights an atypical presentation of subacute thyroiditis and adds a new presentation to the discussion for patients with this condition.

## Introduction

Subacute thyroiditis (SAT) is a rare form of thyroid disease characterized by fever, anterior neck pain, and thyroid hormone abnormalities [1]. SAT affects 12 in 100,000 each year [2]. It is more common in women than men and its incidence decreases with age [2]. SAT is believed to be triggered by a viral infection and has a prodrome of malaise, fatigue, and myalgias. However, it is not the virus itself but rather the post-viral inflammation that causes the cytotoxic T-cell recognition of viral and cell antigens in the thyroid gland, leading to thyroid dysfunction. This inflammation causes the thyroid follicles to be infiltrated with inflammatory cells that disrupt the basement membrane and release stored thyroid hormone into the blood [3].

SAT has three hallmark phases: thyrotoxicosis, hypothyroidism, and euthyroid. Upon initial destruction of the thyroid follicles, patients present with symptoms of hyperthyroidism such as palpitations, weight loss, heat intolerance, and diaphoresis [4]. This phase typically peaks at one week, as the stored thyroid hormone is depleted [5]. Next, patients experience symptoms of hypothyroidism, such as fatigue, myalgias, constipation, and weight gain. This stage can range from weeks to months, as the follicular fibrosis heals and the hypothalamic pituitary thyroid axis adjusts. While most patients return to the euthyroid state after the resolution of their symptoms, up to 25 percent of patients may remain permanently hypothyroid [2,4]. The likelihood of permanent hypothyroidism is not correlated with the severity of SAT [4]. Although it is rare, up to 20% of patients can experience recurrence of SAT [4,6].

The diagnosis of SAT is made clinically. Patients must have a tender thyroid gland on physical exam and abnormalities in their thyroid hormone levels. Upon initial presentation of thyroid pain, most patients exhibit signs of thyrotoxicosis and have an elevated free thyroxine (fT4) and a suppressed thyroid stimulating hormone (TSH). During the hypothyroid, or thyroid burnout phase, patients have an elevated TSH and a low fT4. On ultrasound, SAT can be differentiated from other hyperthyroid disorders, such as Graves’ disease, by its decreased blood flow. It can also be differentiated from malignancy by its areas of hypoechogenicity and lack of irregular, jagged margins [7,8]. However, imaging is not the standard of care and should be reserved to aid in the diagnosis of unclear clinical pictures. A diagnosis of SAT can be made quickly with physical exam, thyroid function tests, and history.

The goal of SAT treatment is to improve symptoms and prevent recurrence. Medication choice depends on the severity of symptoms. Non-steroidal anti-inflammatory drugs (NSAIDs) are recommended as a first-line therapy for mildly symptomatic patients and oral steroids such as prednisone can be added if symptoms worsen. Patients with moderate to severe symptoms should be started on oral steroids primarily, as steroids have been shown to provide symptomatic relief within 24 hours of initiation. The American Thyroid Association recommends 40mg/day of oral steroids for the treatment of moderate to severe SAT [9].

Intrathyroid steroid injections are also an option for the treatment of painful SAT. In the 2021 study by Forkert et al. comparing steroid injections to the standard of care, oral prednisone, the use of intrathyroid steroid injections was found to provide more rapid pain relief and maintain remission. Additionally, patients who received an intrathyroid steroid injection had fewer systemic side effects such as insulin intolerance, weight gain, and menstrual irregularities, as opposed to patients on an oral steroid pack [2]. Currently, there is no medication to prevent permanent hypothyroidism. However, oral steroid therapy with a slow taper has been shown to prevent recurrence [9].

In addition to thyroid pain, many patients with SAT experience severe symptoms of hyper and hypothyroidism. For thyrotoxicosis symptoms, patients may be treated with beta blockers such as propranolol. Thioamides like methimazole and propylthiouracil are not recommended as they inhibit thyroid peroxidase, reducing the production of thyroid hormone. This does not aid in SAT, as thyroid hormone production is not increased; rather, the thyroid follicle with stored hormone is lysed releasing excess hormone into the blood. Patients experiencing symptoms of hypothyroidism in the late phase of SAT may be treated with levothyroxine and titrated according to their symptoms [2].

## Case presentation

A 34-year-old female presented to the clinic complaining of neck pain and a headache for the past two days. She endorsed fatigue, myalgias, dizziness, and constipation. She denied any fever or cough. She reported that she took some ibuprofen but only received minimal pain relief. The patient denied any recent illness but reported that she had been sick with symptoms she attributed to allergies intermittently for the past few months. The patient is a mother to young children, so this was not unusual for her. Her past medical history was non-contributory. She was not currently taking any medications and she was not allergic to any medications. She denied any use of tobacco or vape products. Her only surgical procedure was a loop electrosurgical excision procedure in 2018 for a low-grade squamous epithelial lesion. Her family history was positive for a maternal aunt with thyroid cancer that was treated with surgery. 

Upon exam, the patient’s vital signs were all within normal limits. Her temperature was 98.1 degrees Fahrenheit. Her blood pressure was 117/81 with a heart rate of 84. Her respiratory rate was 16 and her oxygen saturation was at 99% on room air. The patient’s physical exam was notable for neck tenderness over the right lobe and isthmus of the thyroid, thyromegaly, and a palpable thyroid nodule. The remainder of her physical exam, including the pulmonary, cardiac, and gastrointestinal systems, was unremarkable. At this time, our differential diagnosis included mononucleosis, pharyngitis, oropharyngeal abscess, Hashimoto’s thyroiditis, Grave’s disease, post-partum thyroiditis, SAT, thyroid malignancy, toxic adenoma, struma ovarii, painless thyroiditis, and exogenous thyroid hormone use. 

To determine the etiology of the patient’s thyromegaly, we ordered a complete blood count (CBC), TSH, and fT4. The patient’s CBC was notable for a decreased hemoglobin level of 11.1, a decreased hematocrit level of 34.5, and a slightly elevated platelet count of 422. Her white blood cells (WBCs), red blood cells (RBCs), mean corpuscular volume (MCV), mean corpuscular hemoglobin (MCH), mean corpuscular hemoglobin concentration (MCHC), red cell distribution width (RDW), neutrophils, lymphocytes, monocytes, eosinophils, and basophils were all within normal limits. However, her thyroid study came back as abnormal. Her TSH was elevated and her fT4 was severely decreased (Table 1). According to her thyroid results, our patient was in a hypothyroid state.

**Table 1:** Initial Thyroid Studies TSH: Thyroid stimulating hormone, fT4: free thyroxine

Hormone	Measured Value	Reference Range
TSH	2.1 mIU/L	0.8 - 1.8 mIU/L
fT4	<0.01ng/dL	0.4 - 4.5 ng/dL

Because of the patient’s normal vital signs and normal WBC, we were able to rule out infectious etiologies such as mononucleosis, pharyngitis, and oropharyngeal abscess. Additionally, due to her hypothyroid state, past medical history, and physical exam, we were able to rule out Grave’s disease, thyroid malignancy, toxic adenoma, struma ovarii, painless thyroiditis, and exogenous thyroid hormone use. Due to the patient’s hypothyroid state and painful thyromegaly, we were able to confidently make the diagnosis of SAT, or De Quervain’s thyroiditis. 

At the time of initial diagnosis, we started the patient on ibuprofen 600mg every six hours by mouth to see if the pain decreased. On day three, the patient returned for a follow-up visit and expressed persistent neck pain with swallowing and rotation of her neck. Due to these new symptoms, we started her on 40 mg prednisone for two weeks. At her two-week follow-up, the patient reported that she had pain relief with the prednisone, but her symptoms returned after completing her medication. She was subsequently placed on a steroid taper with control of her pain.

## Discussion

SAT can develop days to weeks after viral infections like SARS-CoV-2 and present with neck pain and fever. Development of SAT is not a reflection of the severity of the virus. While our patient denied any history of recent illness, patients with SARS-CoV-2 can be asymptomatic. Our patient could have had community exposure to this virus and developed SAT later. In addition to viral infection, there have also been cases reported of SAT occurring in healthy patients after the administration of the COVID-19 mRNA vaccine. It is proposed that the SARS-CoV-2 spike protein and thyroid antigens exhibit cross-reactivity, creating an inflammatory environment leading to SAT [10]. 

Most patients with symptomatic SAT are found to be in a hyperthyroid state and have thyroid pain on initial presentation [4]. However, our patient deviated from this norm, as she had acute thyroid pain for two days in a hypothyroid state. The mechanism of thyroid pain in SAT is not well described in the literature. It is likely due to the inflammation and active destruction of thyroid follicles. After the initial inflammation in the hyperthyroid state, thyroid pain typically decreases. Most of the thyroid hormone fluctuations caused by SAT peaked one week after the onset of symptoms [5]. However, our patient reported thyroid pain and symptoms of fatigue, myalgias, and constipation consistent with hypothyroidism. Her lab work reflected this diagnosis. 

To our knowledge, there have been no reported cases of patients presenting initially with SAT in the hypothyroid state. One possible theory is that our patient developed SAT post-virally, had rapid efflux of thyroid hormone, became profoundly hypothyroid, and did not notice her symptoms of hyperthyroidism or was asymptomatic. Another proposal is that our patient had a genetic predisposition to SAT, making her more likely to become symptomatic and require treatment. It is well established that the HLA-Bw35 antigen is associated with symptomatic SAT [11]. This could also have accounted for her unique presentation.

SAT is not common in our patient population and adds a new presentation to the differential diagnosis for patients presenting with neck pain and hypothyroid symptoms. This is particularly important in an area where SARS-CoV-2 is now endemic. While we have no precise origin of our patient’s SAT, we can suspect that she likely encountered a virus in the community, leading to a unique inflammatory presentation.

## Conclusions

SAT is a rare presentation of thyroid disease that most often presents with neck pain, fever, and thyrotoxicosis. However, it may present initially with neck pain and symptomatic hypothyroidism, as in our patient described above. This case highlights an atypical presentation of SAT that is important to remember in the differential of neck pain.
